# Supplementary material for: Evaluation of an Adapted Semi-Automated DNA Extraction for Human Salivary Shotgun Metagenomics
Source: Biomolecules. 2023 Oct 11;13(10):1505. doi: 10.3390/biom13101505 (PMC10604855; doi:10.3390/biom13101505)
Supplement: Supplementary file 1 [file biomolecules-13-01505-s001.zip › Supplementary-Figures.pdf]

Supplementary Information

Evaluation of an Adapted Semi-Automated DNA Extraction for Human Salivary Shotgun Metagenomics

Victoria Meslier [1,2,†], Elisa Menozzi [2,3,†], Aymeric David [1,2,†], Christian Morabito [1,2], Sara Lucas Del Pozo [2,3],
Alexandre Famechon [1,2], Janet North [4], Benoit Quinquis [1,2], Sofia Koletsi [2,3], Jane Macnaughtan [2,5], Roxana Mezabrovschi [2,3], S. Dusko Ehrlich [2,3], Anthony H. V. Schapira [2,3,*] and Mathieu Almeida [1,2,*]

[1] MetaGenoPolis, INRAE, Université Paris-Saclay, 78350 Jouy-en-Josas, France

[2] Aligning Science Across Parkinson's (ASAP) Collaborative Research Network, Chevy Chase, MD 20815, USA

[3] Department of Clinical and Movement Neurosciences, UCL Queen Square Institute of Neurology, University College London (UCL), London WC1E 6BT, UK

[4] Research Department of Hematology, Cancer Institute, University College London (UCL), London WC1E 6BT, UK

[5] Liver Failure Group, Institute for Liver and Digestive Health, University College London, London WC1E 6BT, UK

* Correspondence: a.schapira@ucl.ac.uk (A.H.V.S.); mathieu.almeida@inrae.fr (M.A.)

† These authors contributed equally to this work.

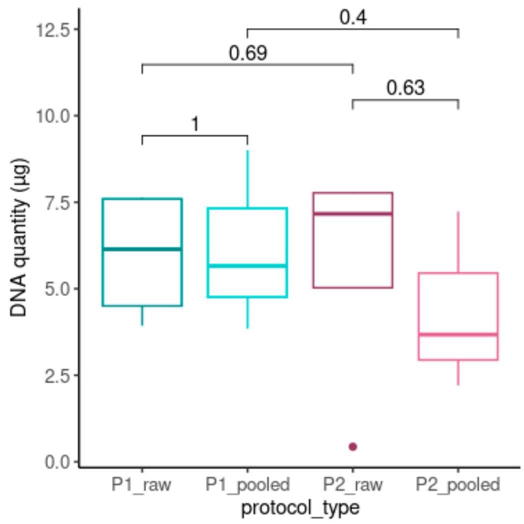

Figure S1: Effect of DNA isolation on DNA quantity (µg) per protocol and sample type. P-values from unpaired Wilcoxon tests are reported.

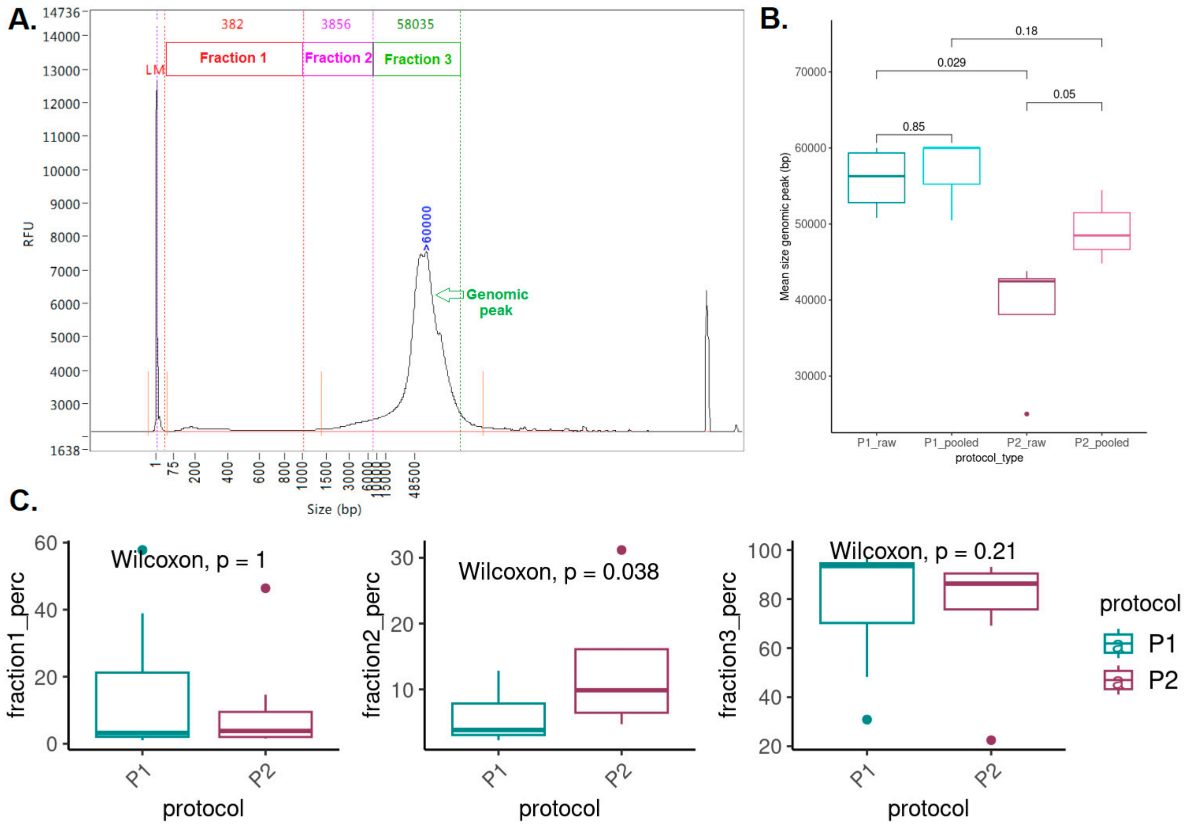

Figure S2: Overall assessment of the DNA quality. A. Typical DNA profile obtained from Fragment Analyzer, measuring the quality of the DNA after extraction. Three fractions can be defined depending on the distribution of the size of the DNA: fraction 1, fragments ≤ 1 000bp; fraction 2, >1 000bp and ≤10 000bp and fraction 3 >10 000bp. **B.** Mean size of the genomic peak per protocol and sample type. **C.** The area under the fragment analyzer curve was determined for each fraction 1 to 3 for each sample and reported as percentage of the total area under the curve. P-values from unpaired Wilcoxon tests were reported.

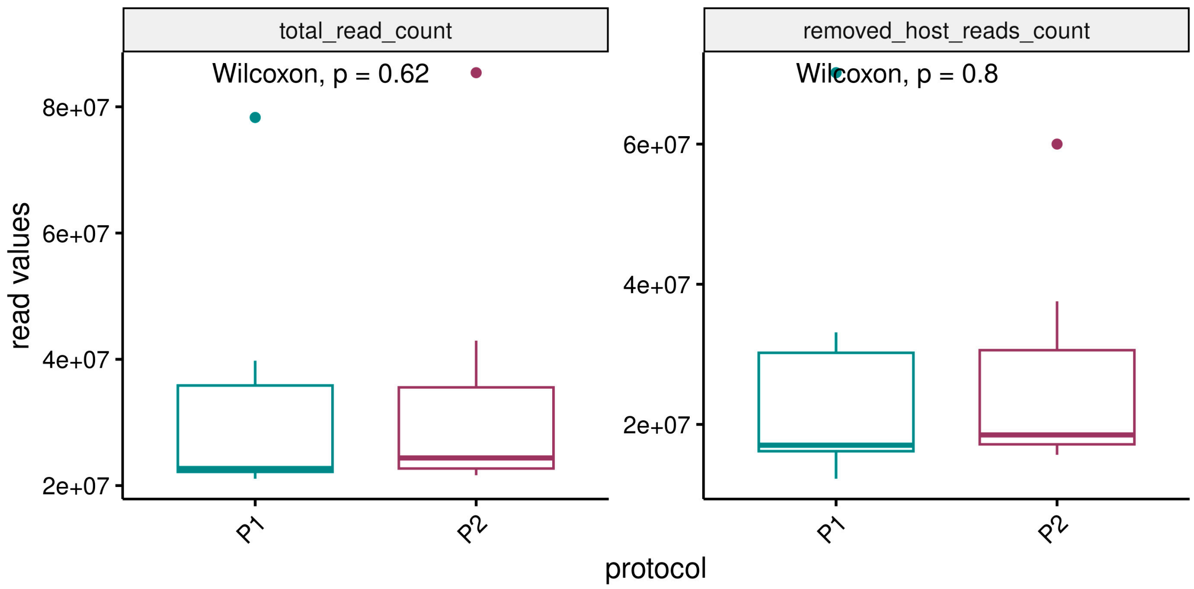

Figure S3: Whole Shotgun Sequencing report. The total number of raw reads, the number of human related reads and the number of High Quality (HQ) cleaned reads, corresponding to reads cleaned from low quality and human reads, were used as indicators of WGS sequencing performances.

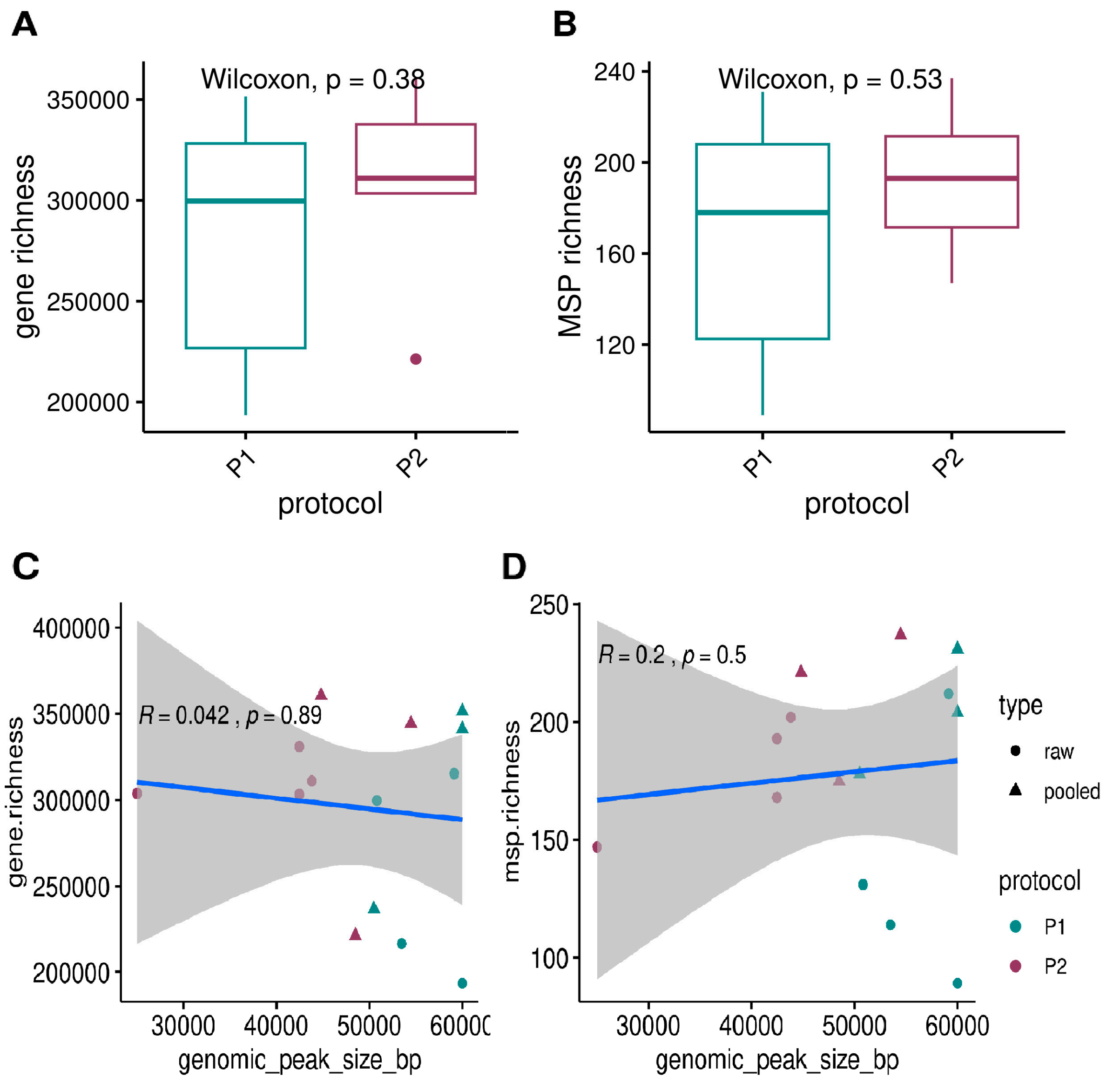

Figure S4: Additional comparison for alpha diversity metrics. **A**. Gene and MSP species richness metrics for all samples per protocol. **B**. Spearman correlation between gene or MSP species richness and the DNA quality using the size of the genomic peak. Samples were color coded by protocol and shaped by sample type.

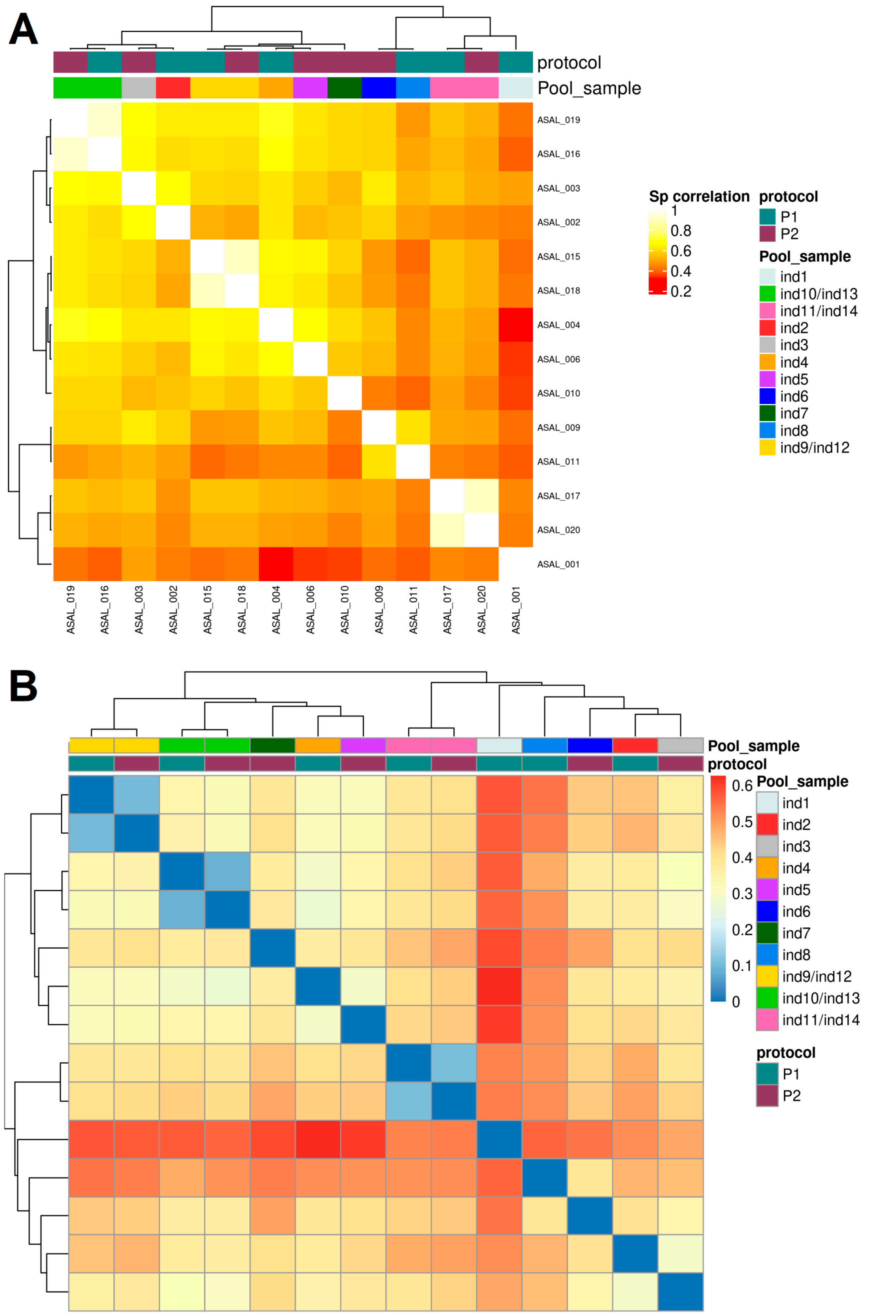

Figure S5: Heatmaps based on MSP species composition. **A**. Spearman correlations. **B**. Bray-Curtis dissimilarity. Protocols and Pool_sample ID are reported.
